# Postural lung recruitment assessed by lung ultrasound in mechanically ventilated children

**DOI:** 10.1186/s13089-017-0073-0

**Published:** 2017-10-13

**Authors:** Gerardo Tusman, Cecilia M. Acosta, Stephan H. Böhm, Andreas D. Waldmann, Carlos Ferrando, Manuel Perez Marquez, Fernando Suarez Sipmann

**Affiliations:** 1 Department of Anesthesiology, Hospital Privado de Comunidad, 7600 Mar del Plata, Buenos Aires Argentina; 2 Hepa Wash GmbH, Munich, Germany; 3 Swisstom AG, Landquart, Switzerland; 4 Department of Anesthesiology, University Hospital Valencia, Valencia, Spain; 5 Department of Intensive Care Medicine, Instituto de Investigación Sanitaria Fundación Jiménez Díaz, IIS-FJD, Madrid, Spain; 6 Hedenstierna Laboratory, Department of Surgical Sciences, Section of Anesthesia and Critical Care, Uppsala University Hospital, Uppsala, Sweden; 7 CIBERES, Madrid, Spain; 8 Department of Critical Care, Hospital La Fe, Valencia, Spain

**Keywords:** Children, Airways, Outcome, Respiration, Lung ultrasound, PEEP, Anesthesia-induced atelectasis, Lung recruitment, Mechanical ventilation

## Abstract

**Background:**

Atelectasis is a common finding in mechanically ventilated children with healthy lungs. This lung collapse cannot be overcome using standard levels of positive end-expiratory pressure (PEEP) and thus for only individualized lung recruitment maneuvers lead to satisfactory therapeutic results. In this short communication, we demonstrate by lung ultrasound images (LUS) the effect of a postural recruitment maneuver (P-RM, i.e., a ventilatory strategy aimed at reaerating atelectasis by changing body position under constant ventilation).

**Results:**

Data was collected in the operating room of the Hospital Privado de Comunidad, Mar del Plata, Argentina. Three anesthetized children undergoing mechanical ventilation at constant settings were sequentially subjected to the following two maneuvers: (1) *PEEP trial* in the supine position PEEP was increased to 10 cmH_2_O for 3 min and then decreased to back to baseline. (2) *P*-*RM* patient position was changed from supine to the left and then to the right lateral position for 90 s each before returning to supine. The total P-RM procedure took approximately 3 min. LUS in the supine position showed similar atelectasis before and after the PEEP trial. Contrarily, atelectasis disappeared in the non-dependent lung when patients were placed in the lateral positions. Both lungs remained atelectasis free even after returning to the supine position.

**Conclusions:**

We provide LUS images that illustrate the concept and effects of postural recruitment in children. This maneuver has the advantage of achieving recruitment effects without the need to elevate airways pressures.

**Electronic supplementary material:**

The online version of this article (doi:10.1186/s13089-017-0073-0) contains supplementary material, which is available to authorized users.

## Background

The incidence of atelectasis in mechanically ventilated children with healthy lungs is as high as 68–100% [1–4]. Anesthesia-induced atelectasis has been well described by different imaging techniques such as CT, MRI and lung ultrasound (LUS) [1–5]. The main mechanism leading to atelectasis formation during anesthesia is a decrease in functional residual capacity due to lung compression through the dysfunctional diaphragm by abdominal content [6, 7]. During such conditions, the trans-pulmonary pressure (Ptp = airways − pleural pressure) is no longer sufficient to offset these compressive forces on the most gravity-dependent parts of the lungs, where Ptp is naturally the lowest. *Compression atelectasis* has clearly been demonstrated in anesthetized children and adults [3, 7–10].

### Rationale of postural lung recruitment

Lung recruitment maneuvers are ventilator strategies that elevate airway pressures for a few breaths to reaerate atelectasis [11, 12]. Such maneuvers are safe and easily to conduct in mechanically ventilated children [3, 13]. However, potential hemodynamic side effects of high intrathoracic pressures necessitate close hemodynamic monitoring and that such maneuvers are performed in normovolemic patients, only [12].

In this short communication, we explain the rationale of a new type of lung recruitment maneuver—the postural recruitment (P-RM). It can be defined as an active reaeration of atelectatic lung tissue by intentional changes of a patient’s body position under constant ventilatory conditions. This concept makes use of the influence that gravity has on respiratory physiology, more precisely on Ptp. The superimposed pressure within the thorax caused by the lung’s own weight decreases Ptp by approximately 0.25 cmH_2_O for every 1 cm of ventral-to-dorsal thoracic diameter. Therefore, in supine mechanically ventilated patients Ptp decreases along the gravitational axis causing the lungs to collapse in their most dorsal parts (lowest Ptp) while keeping the ventral zones (highest Ptp) aerated and thus “open” during the entire respiratory cycle [12]. No matter in which body position the patient is placed, dorsal lung zones will always be prone to atelectasis and airways closure [6, 7, 10].

Considering this gravity-dependent physiology, the additive effect of the two following principles can be used therapeutically to recruit lungs by intentionally changing the body position of ventilated patients:

- The *first principle* refers to the relationship between Ptp and body position. It postulates that collapsed tissue in dependent lung zones can be recruited by placing the patient in the opposite position (i.e., from supine to prone or from the left to the right lateral decubitus position). This way, previously collapse lung tissue is now located in the non-dependent position where it is being subjected to the expanding forces of higher Ptps.
- The *second principle* refers to the stabilizing effect of positive end-expiratory pressure (PEEP) on airways and alveoli. It postulates that—provided sufficient PEEP is applied—well ventilated and fully aerated non-dependent lung areas will remain “open” even when becoming dependent again after returning the body to its initial position.

**Fig. 1:**
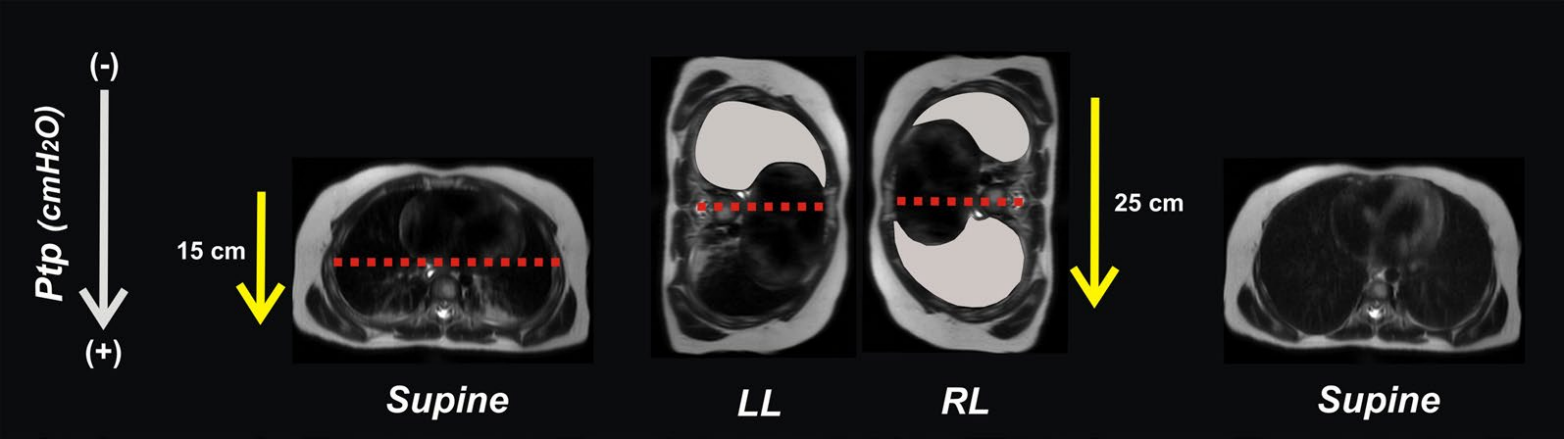
Concept of postural lung recruitment. Theoretical explanations of the postural recruitment maneuver in an anesthetized child 6 years of age. The maneuver consists of sequential changes in body position from supine to both lateral positions and back to supine again, keeping ventilatory settings constant. The gradient of trans-pulmonary pressures (Ptp) differs between body positions due to the elliptical shape of the chest with the gravity-dependent thoracic dimension being larger in lateral than in the supine position (yellow arrows). Thus, the lower half of the lungs is predisposed to collapse while the upper half is usually aerated and “open” during the entire respiratory cycle (red dotted line). In the left lateral position (LL), the entire right upper lung has the chance to open up at a low plateau pressures even under standard ventilator settings (gray lung). Once open, this right lung can maintain its “open lung” condition when turned to the right lateral positioning (RL) provided sufficient PEEP is applied. Notice the larger vertical distance in the lateral position required a higher PEEP to counteract the potential decrease in Ptp in the dependent lung. Now, the left lung is being recruited as it is placed in the uppermost gravity non-dependent position. At the end of the postural recruitment maneuver both lungs are free from atelectasis although the patient has returned to the baseline supine position

Based on the above principles, we propose a postural recruitment maneuver in which a supine patient is first turned onto his left, then onto his right side and finally to his original supine position (Fig. 1). Thus, we speculate that lung recruitment can be achieved by postural changes without having to elevate airway pressures [11, 12].

Mechanically ventilated children are a sub-population that could especially benefit from this intervention for two reasons: (1) the respiratory physiology of pediatric patients makes them more susceptible to lung collapse after tracheal intubation than adults [8, 9], and (2) children are usually of light weight and can thus be easily turned by one single operator.

In this short communication, we present lung ultrasound (LUS) images which confirm the recruitment effect of intentional changes of body position in anesthetized children. LUS is a good non-invasive tool for monitoring lung aeration during and after lung recruitment maneuvers in real-time fashion [14–16].

## Effects of postural lung recruitment

We analyzed three anesthetized children undergoing open inguinal hernia repair. Children aged 12, 24 and 34 months weighing 10, 12 and 18 kg, respectively. After approval by the local Ethical Committee and after obtaining Informed Consent from the parents, patients received general anesthesia under standard monitoring. Their lungs were ventilated in a pressure-controlled mode using an Aespire 7900 (Datex-Ohmeda, GE Healthcare, Helsinki, Finland) with the inspiratory pressure set to achieve a tidal volume of 7 mL/kg (approx. 12 cmH_2_O) and a respiratory rate adjusted between 20 and 25 bpm to keep end-tidal of carbon dioxide between 35 and 40 mmHg. PEEP was 5 cmH_2_O, I:E 1:1 and FIO_2_ 0.5.

**Fig. 2:**
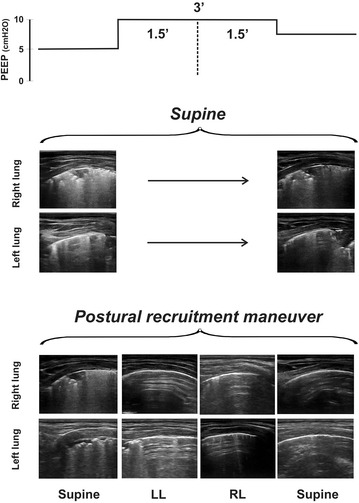
Lung ultrasound images of postural recruitment. Example of the postural recruitment effect in one anesthetized child (case 1–24 months). This patient was subjected to 10 cmH_2_O of positive end-expiratory pressure (PEEP) in the supine position and then during the postural recruitment maneuver. Bilateral atelectasis was diagnosed placing the ultrasound linear probe in the oblique position over the juxta-diaphragmatic lung areas. The same pulmonary areas were assessed in each body position. Note that atelectatic areas are reaerated only after the postural changes

**Fig. 3:**
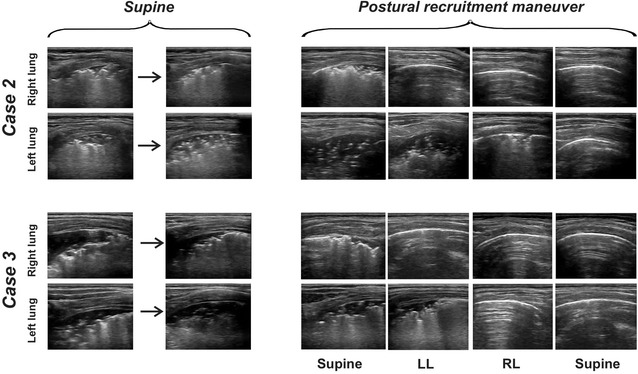
Lung ultrasound images of postural recruitment in case 2 and 3

Lung aeration was assessed by LUS using the linear 7–12 MHz transducer of the Esaote (MyLab Gamma, Genova, Italy). After anesthesia induction a standard bilateral LUS examination was performed in the supine position. Thereafter the echo probe was placed in an oblique direction between the ribs in the most dependent position as previously described [5]. All patients presented subpleural consolidations, air bronchograms and B-lines as signs of atelectasis (Figs. 2, 3).

Each patient was subjected to the following protocol sequence (Fig. 2):

- *Testing the reaerating effect of 10 cmH*_2_ *O of PEEP* After anesthesia induction in the supine position, PEEP was increased from 5 to 10 cmH_2_O keeping ventilation constant for 3 min. Then, ventilation returned to baseline settings but keeping PEEP at 8 cmH_2_O to maintain any potential recruitment that 10 cmH_2_O of PEEP may have induced.
- *Testing the effect of postural recruitment* Before emergence from anesthesia, patients were placed in the left lateral decubitus position for 90 s at constant ventilation but raising PEEP to 10 cmH_2_O. Thereafter, patients were turned on their right side for another 90 s. At the end of the 3rd min, patients were placed back in the supine position returning to baseline ventilation, however, at a PEEP of 8 cmH_2_O to keep the lungs open.

Figure 2 depicts the results of the protocol in one patient (case 1–24 months). LUS images taken at 8 cmH_2_O of PEEP show that 3 min of ventilation with 10 cmH_2_O of PEEP in the supine position did not have any measurable recruitment effect. Atelectasis persisted during and after PEEP elevation in all patients. Figure 3 shows the effect of the P-RM in the other two patients (case 2–12 months and case 3–34 months). The Additional file 1: video S1, Additional file 2: video S2 and Additional file 3: video S3 show the corresponding results than Figs. 2 and 3.

By contrast, the changing body positions of the P-RM clearly restored lung aeration in the atelectatic areas at the same ventilator settings. Reaeration of previously collapsed dependent lung areas was always observed as soon as they became non-dependent in the uppermost position; first in the right lung with the patient lying in the left lateral position and then in the left one with the patient in the right lateral decubitus. After the P-RM, both lungs remained free from atelectasis in the final supine position (Figs. 2, 3; Additional file 1: video S1, Additional file 2: video S2 and Additional file 3: video S3). Hemodynamics maintained stable throughout the protocol.

## Commentary

We present LUS images documenting the effects of postural lung recruitment in children. Sequential changes in body position during constant ventilation at PEEP 10 cmH_2_O decreased atelectasis even without applying the high airways pressures of classical recruitment maneuvers.

The therapeutic effect of P-RM seems to result from an increased Ptp caused by a gravity-dependent decrease in pleural pressure in the dorsal lungs and not by elevated airway pressures. As the right-to-left thoracic diameter is much larger than the anterior–posterior one, when a patient is placed in the lateral position, the resulting inspiratory Ptp within the uppermost lung will increase reaching the effective opening pressure of the collapsed lung tissue located in this region (Fig. 1). After the P-RM, a PEEP of 8 cmH_2_O was enough to maintain an “open lung” condition in these three healthy patients [11].

The therapeutic implications of P-RM can be derived from the known negative consequence that atelectasis have on mechanically ventilated lungs. Perioperative respiratory complications such as hypoxemic events, bacterial translocation and pneumonia [17–21] adversely affect patient’s outcomes while increasing health care cost in both, adults and pediatric patients [22, 23]. Lung recruitment maneuvers easily overcome atelectasis in children with healthy and sick lungs alike [3, 13]. They quickly abort hypoxemic events and could have some protective effect in atelectasis-mediated pneumonias [17, 24, 25].

However, the main objections against recruitment maneuvers are related to their potential hemodynamic side effects, especially in children. Reflexes triggered by high intrathoracic pressures cause bradycardia, compression of pulmonary capillaries and reduced venous return. Each of these either acting in isolation or combined are responsible for the hemodynamic impairment frequently encountered during recruitment maneuvers, predominantly in hypovolemic patients. In view of such undesirable consequences, the P-RM introduced in this paper presents an effective and efficient low risk alternative intervention for treating atelectasis in children. Turning the patients while applying 10 cmH_2_O of PEEP for just 3 min seems to be uncritical in normovolemic children.

As far as we know, a postural lung recruitment maneuver has never before been described in children, although we found studies related to the effect of body positioning on respiratory function of mechanically ventilated patients. Some studies describe positive effects of lateral and prone positioning on respiratory function [26, 27] while others do not [28–30]. In these studies, body position was changed leaving ventilatory settings and PEEP constant. The lack of adequate PEEP adaptations—an essential therapeutic intervention of any recruitment strategy such as ours—may have led to these contradictory results. We reasoned that a slightly elevated PEEP should be applied for two reasons: (1) to obtain a plateau pressure high enough to reach the opening pressure of the uppermost lung areas and (2) to maintain the new recruited regions open when returning the patient to the supine position.

## Conclusions

In this short communication, we present LUS images documenting the positive effects of postural recruitment in children. Changes in body position during constant ventilation at 10 cmH_2_O of PEEP decreased atelectasis without the need to elevate airway pressures as in conventional recruitment maneuvers. Bedside imaging techniques such as LUS can help identify the best settings for a P-RM adapting them to the individual patient.

## Additional files

**Additional file 1: Video S1.** Lung ultrasound images (LUS) of the isolated effect of 10 cmH_2_O of PEEP and postural recruitment maneuver—case 1.

**Additional file 2: Video S2.** Lung ultrasound images (LUS) of the isolated effect of 10 cmH_2_O of PEEP and postural recruitment maneuver—case 2.

**Additional file 3: Video S3.** Lung ultrasound images (LUS) of the isolated effect of 10 cmH_2_O of PEEP and postural recruitment maneuver—case 3.
